# Enlargement of the Spleen

**Published:** 1858-03

**Authors:** J. W. Crooks

**Affiliations:** Rockport, Indiana


ARTICLE IV.

### ENLARGEMENT OF THE SPLEEN.

BY J. W. CROOKS, M. D., ROCKPORT, INDIANA.

I have reason to believe that there is not that professional importance attached to the varied derangement of this organ that the subject justly demands. The spleen is liable to various forms of disease; among the most frequent is, that which is thought to be a sequel to our western autumnal remitting and intermitting fevers. In malarious districts, the observing practitioner has not failed to notice the fact, that derangements of this organ play a conspicuous part in most maladies to which his attention is solicited. The position and close relation of the spleen to important organs, render any manifest deviation from the standard of health quite improbable, without affecting more or less their normal functions. True, the special function of the spleen is not even *probably* understood; yet, its diseased condition is soon rendered apparent by manifest disturbances of a special and general character. Though, as to itself, it does seem that, if it had a territorial area in proportion to its varied mutations, it might carry on its mysterious workings with but little disturbance to the general health. But its relative situation is such, that upon any considerable increase of size, its encroachment upon neighboring organs cannot be borne with impunity; hence, the evil effects of enlarged spleen are more apparent upon adjacent parts than upon its own substance. This fact, doubtless, arises from the nature of its anatomical structure. Being composed almost entirely of blood-vessels, and but sparingly supplied with nerves, it yields to any amount of distention, with little sensibility to its own body; but when in a state of hypertrophy or engorgement with induration, it cannot fail to produce both functional and organic disturbances, often of a serious character. I have witnessed instances of its materially impeding respiration by its encroachment upon the diaphragm; indigestion, and other derangements of the stomach, from the same cause; and from its weight and compression upon the bowels, constipation and difficult defecation. The great vessels may be compressed too, causing an unequal distribution of the blood—some organs receiving too much, others not enough—thus giving rise to various forms of internal congestions, dropsical effusions, with all their kindred affections, resulting from impediments in the circulation.

Though enlarged spleen is often a sequel to our western remitting and intermitting fevers, it is by no means an invariable attendant. And though ague frequently precedes the development of the condition in question, it often arises without being accompanied with any form of fever. The idea that the cold stage of intermittents is the principal and predominant cause of "ague cake," is one that I respectfully decline subscribing to. I have witnessed too many cases of enlarged spleen, in no way directly connected with any variety or form of fever, not to apprehend some more potent agency in their production. And I would rather believe, that marsh miasm acts, firstly, upon the spleen; secondly, upon the system, in causing chill and fever; that the chill is the result of the engorged spleen, than that the affection of the spleen the result of the chill. That the spleen is peculiarly susceptible to the influence of malaria, more so than any other organ, the liver not excepted, I am fully satisfied. Congestions and enlargements of the body are often the first indications we have of an approaching chill and fever; and, in fact, this condition may exist for considerable time totally unawares to the patient, and unattended with either chill or fever. Its great want of sensibility disqualifies it from sounding the first note of alarm, when its own individual substance is the lone theatre of disease. In malarious localities, patients frequently present themselves, complaining of a dull sort of uneasiness in the left hypochondrium, extending to the precordial region—not having suffered from chills or any form of fever, have no suspicion of enlarged spleen, but, on examination, this latter condition is found to be the true source of all the trouble. And it is not uncommon for this state of affairs to continue for many months, or even years, alternately diminishing and increasing, but never entirely disappearing, and the patient at the same time totally exempt from chill and fever.

Then, when we take into consideration the fact, that an enlargement of the spleen often occurs and persists for months and for years without ague, and that it as often precedes the febrile attack when they are connected at all, as it seems to follow as a sequel, it is not unreasonable to conclude, that derangements of the spleen have as much to do in producing ague as the latter has in producing the former. Then, that character of enlargement of the spleen, known as "ague cake," I am disposed to regard as a true idiopathic affection, incited and maintained by a direct miasmal influence—and the principal function of the spleen to be a sort of depurator to the system, a sort of cesspool or reservoir for the noxious properties of the system, which, by process of time and some pecular action, it neutralizes the poisonous influence. There is nothing improbable, but, on the other hand, an apparent wise provision of nature, that the system should be provided with a diverticulum and a regenerating laboratory, through which deleterious agents might pass, modified, and rendered innoxious to the general economy. That it possesses these qualities to a considerable extent, and often, as it were, flies to the rescue of the more vital organs, is neither unreasonable nor scarcely doubtful.

*Treatment.*—That calomel acts specifically upon the glandular system in general, and more particularly the liver, we have but little if any doubt. And this agent was once the Samson remedy for all forms of hepatic and biliary derangements of every character. Calomel, portal congestion, and black bile, were "all the go." And the spleen, too, though deprived of most of the characteristics of a gland, having no excretory duct through which vitiated matter could pass, was required to perform similar functions with the liver.

Then, have we anything that will act specifically on the spleen when in the condition under consideration? I answer we have. For that form of disease, resulting from the influence of malaria, we have what I regard an infallible remedy in the sulph. quinine. By its specific and direct influence upon the spleen, through the medium of the blood, it neutralizes the poisonous effects of malaria, and at once removes the whole cause of the trouble. The effects of quinine in neutralizing, or otherwise counteracting the effects of this source of disease, is perfectly apparent in the treatment of ague and fever, as well as other biliary derangements of the system. We do not give calomel now-a-days for the purpose of arresting bilious fever; nor do we give mercury in the treatment of ague, or, at least, very rarely, and I venture to say, never any more with reference to its mercurial effect. The condition of the spleen in question being the result of miasmal influence, quinine is the remedy, as its known effects upon this class of morbific agents are now placed beyond question. With this remedy alone, I treat chill and fever and enlargements of the spleen, premising purgation only with reference to the condition of the bowels. And I here bear testimony to the curative effects of quinine in all those forms of summer and autumnal *diarrhœa* so prevalent amongst children, more particularly at those seasons of the year. The constitution of a child seems to be such, that it cannot tolerate the accumulative influence of malaria; it will soon succumb under some form of the terrific nervous affections, or exhaust itself in the effort to get rid of the pernicious effects of the poison by diarrhœa. Whereas, by the timely and proper use of quinine, and the restraining influence of opiates, many cases may be cured that would prove fatal under the old calomel practice. I regard its modus operandi, in these forms of diseased action, the same as upon the spleen, by at once acting upon the malaria in the system, neutralizing and destroying its effects. About ten grains of quinine daily—grs. iii. at a dose—for from two to six weeks, I have found fully adequate to the relief of the most obstinate and protracted enlargement of the spleen.

I cannot refrain from venturing the suspicion, that actual hepatitis and other formidable diseases of both liver and spleen were more common, and much more ungovernable, under the winged god, Mercury, than at the present time, when many of the profession begin to suspect him for raising his magic wand to

> **"Draw bodies down to hollow graves,**
> **And send them off on Stygian waves."**

